# 

**Published:** 1849-01

**Authors:** 


WHOLE SERIES—VOL. V, NO. V.

Vol. I.] DECEMBER 1848, JANUARY, 1849. [No. 5.

### THE NORTH-WESTERN MEDICAL AND SURGICAL JOURNAL.

PUBLISHED EVERY OTHER MONTH.

AT TWO DOLLARS PER ANNUM IN ADVANCE.

EDITED BY

WILLIAM B. HERRICK, M.D, AND JOHN EVANS, M.D.,

PROFESSORS IN RUSH MEDICAL COLLEGE, CHICAGO.

CHICAGO AND INDIANAPOLIS:

PUBLISHED BY JOHN W. DUZAN.

1849.

